# Comparative genomics of endophytic fungi *Apiospora malaysiana* with related ascomycetes indicates adaptation attuned to lifestyle choices with potential sustainable cellulolytic activity

**DOI:** 10.1093/dnares/dsaf011

**Published:** 2025-05-10

**Authors:** Shashi Kant, Sreyashi Das, Subhajeet Dutta, Kajal Mandal, Aditya Upadhyay, Aditya N Sarangi, Rajib Majumder, Sucheta Tripathy

**Affiliations:** Computational Genomics Lab, Structural Biology and Bioinformatics Division, CSIR Indian Institute of Chemical Biology, Kolkata 700032, India; Academy of Scientific and Innovative Research (AcSIR), Ghaziabad 201002, India; Computational Genomics Lab, Structural Biology and Bioinformatics Division, CSIR Indian Institute of Chemical Biology, Kolkata 700032, India; Academy of Scientific and Innovative Research (AcSIR), Ghaziabad 201002, India; Computational Genomics Lab, Structural Biology and Bioinformatics Division, CSIR Indian Institute of Chemical Biology, Kolkata 700032, India; Academy of Scientific and Innovative Research (AcSIR), Ghaziabad 201002, India; Computational Genomics Lab, Structural Biology and Bioinformatics Division, CSIR Indian Institute of Chemical Biology, Kolkata 700032, India; Academy of Scientific and Innovative Research (AcSIR), Ghaziabad 201002, India; Computational Genomics Lab, Structural Biology and Bioinformatics Division, CSIR Indian Institute of Chemical Biology, Kolkata 700032, India; Academy of Scientific and Innovative Research (AcSIR), Ghaziabad 201002, India; Computational Genomics Lab, Structural Biology and Bioinformatics Division, CSIR Indian Institute of Chemical Biology, Kolkata 700032, India; Department of Biotechnology, School of Life Science and Biotechnology, Adamas University, Kolkata 700126, India; Computational Genomics Lab, Structural Biology and Bioinformatics Division, CSIR Indian Institute of Chemical Biology, Kolkata 700032, India; Academy of Scientific and Innovative Research (AcSIR), Ghaziabad 201002, India

**Keywords:** endophytic fungi, *Apiospora*, genome compartmentalization, comparative genomics, cellulase

## Abstract

Ascomycetes fungi produce carbohydrate-active enzymes that are prized in the biofuel industry. Comparative genome analysis of endophytic fungus *Apiospora malaysiana* with seven other closely related high quality genomes of endophytic and pathogenic organisms reveal that effectors and pathogenicity-related genes are predominantly localized within rapidly evolving gene-sparse regions rather than in the conserved region. This suggests bipartite genome architecture where the rapidly evolving region plays a role in host adaptation. Endophytic fungi adapt to plant invasion by enriching enzymes that degrade cellulose, hemicellulose, lignin, and pectin. In contrast, we observed that pathogenic fungi, especially *N. oryzae*, show a reduced number of secondary metabolites biosynthesis and catabolic genes, reflecting lifestyle adaptation. The presence of exclusive sporulating gene clusters in pathogen species could possibly indicate their pathogenic affiliation. Limited genome plasticity and low heterozygosity in *A. malaysiana* are in line with its predominant asexual life cycle choices in lab conditions. The secretome of *A. malaysiana* grown in cellulose-only media had more cellulase activities when compared to cultures grown in YPD media. Genes that were differentially up-regulated in cellulose-only media exhibited strong cellulose-degrading activity and genes involved in evading detection by the hosts surveillance system. Successful cloning and expression of selected CAZymes in bacterial expression systems with desirable physicochemical properties highlight the biotechnological potential of *A. malaysiana* for sustainable cellulolytic enzyme production. These findings position endophytes as valuable resources for cellulolytic enzyme research and broader bio-industrial applications.

## Introduction

Ascomycota is one of the most significant fungal taxa that harbor diverse lifestyles. Endophytic Ascomycetes are asymptomatic in nature; therefore, not much data is available compared to their economically important pathogenic cousins.^1^ There is an immediate need to improve endophytic fungi’s genomic resources and establish their relationship with pathogens. Genomes often reflect the lifestyle of the species; for instance, plant pathogenic Ascomycetes may exhibit genome compartmentalization, while others maintain a more uniform genome structure.^2^ To maintain a uniform genome structure, organisms rely on purifying selection to preserve essential genetic functions.^3^

Endophytic ascomycetes have significant applications beyond their ecological roles. They are crucial in producing medically important compounds like antibiotics and have a long history in traditional fermentation processes. Fungi generally secrete extensive enzymes that can degrade complex carbon and nitrogen substrates and degrade organic and inorganic matter, such as lignocelluloses.^4^ In addition, they play a vital role in maintaining microbial ecosystems.^5^ In the biotechnology industry, at least a dozen filamentous fungi produce enzymes commercially.^6^ There are markedly few studies on producing industrial enzymes from endophytes. In this study, intrigued by the fruity smell of a lab-isolated Ascomycetes growing as a contaminant, detailed genomic and transcriptomic studies were carried out to establish the industrial readiness of this fungus. This organism was initially identified as *Arthrinium malaysianum*^7,8^ based on the analysis of ITS sequences. Recently, this species has been christened as *Apiospora malaysiana* (*Arthrinium malaysianum* Basionym: CBS:102053 MycoBank MB837684).^9^*Apiospora* has diverse ecological habitats, although the sexual stage of *A. malaysiana* was never obtained in culture. *Apiospora* and *Arthrinium* exist as two separate lineages with distinct conidia structures. *Apiospora* conidia are generally rounded in the face view and lenticular in the side view. Endophytic fungi, found in plant tissues like roots, stems, leaves, flowers, and seeds, colonize plants without causing harm to them. *Arthrinium sp.*, an endophyte,^10–12^ is rich in secondary metabolites such as terpenoids, alkaloids, and phenolics, making it valuable for various medicinal and pharmaceutical developments.^8,11^ This organism is remarkable in having bioremediation properties, leading to the conversion of toxic isotopes of chromium and lead into its relatively non-toxic cousins.^7^ We extracted crude proteins from standard and cellulose-only cultures, observing significant cellulase activity in the exudates. Whole-genome sequencing and comparative analysis with related species and two obligate pathogens revealed an increased copy number of cellulase classes for degrading complex carbohydrates. Transcriptomics studies highlighted the expression patterns of carbohydrate-degrading genes. We characterized two distinct protein sets with varied physicochemical properties to assess their potential for cloning, expression, and solubility. This work aims to develop bioactive cellulases capable of hydrolyzing cellulose (30-50% of agro-waste) into simple sugars for bioethanol production.^13^

## Materials and methods

### Culture maintenance and microscopy of *A. malaysiana*

Organisms were cultivated in a YPD medium and incubated at 28 °C in the dark with constant agitation at 150 rpm for 72 hours. After incubation, the cultures were harvested and thoroughly washed with autoclaved distilled water to remove residual media. To induce sporulation, the washed cultures were transferred to solid agar plates containing 2% CaCO_3_ and 50% PDA and incubated for 2 to 3 weeks. The resulting mycelia and spores were observed under a light microscope (EVOS XL Imaging System) at 100× magnification. Filamentous mycelia were further examined by fixing them on glass slides, staining them with DAPI, and visualizing them using confocal microscopy.

### Determination of cellulase activity in secreted proteins

The 48-hour culture exudates of *A. malaysiana* grown in YPD media were filtered through nylon cloth. Proteins were precipitated from 100 ml of the filtered exudates using ammonium sulfate (90% saturation, 65.7 g/100 ml) at 4°C at a regular interval of 10 minutes with constant stirring until fully dissolved.^14^ The solution was centrifuged at 28500 g for 1 hour, the supernatant discarded, and the pellet resuspended in 1 ml PBS. Dialysis was performed overnight at 4°C using a 10 kDa membrane with 1 liter of PBS. After centrifuging at 11600 g for 15 minutes, debris was discarded, and the soluble fraction was concentrated 5× using a 10 kDa centrifugal filter unit (Merck) at 2800 g for 10 minutes. Protein concentration was determined by Bradford assay. A carboxymethyl cellulose (CMC) assay was conducted using 42 µl of protein solution with 2% CMC substrate in 0.05 M sodium citrate buffer (pH 4.8) at 50°C for 30 minutes. The reaction was terminated with a DNS reagent, which indicated glucose release through

 color development.^15,16^ The absorbance of the solution is measured at 540 nm and compared with the standard glucose curve. Sigma cellulase, EC 3.2.1.4 from *Penicillium funiculosum*, C-0901 was used as a positive control.

### Species confirmation and whole-genome sequencing

Freshly grown mycelia were washed in autoclaved water and centrifuged at 2800 g for 5 minutes to pellet the cells. The pellet was frozen in liquid nitrogen and ground to a fine powder. Genomic DNA was isolated using the Thermo Scientific GeneJET Plant Genomic DNA Purification Kit, and residual RNA was removed using RNase A. The Internal Transcribed Spacer (ITS) region was amplified with specific primers (forward: 5′-TCCGTAGGTGAACCTGCGG-3′, reverse: 5′TCCTCCGCTTATTGATATG-3′), sequenced, and analyzed using BLAST to identify the fungal species. The genomic DNA, with a 260/280 ratio of 1.74 and concentration of 39 µg/ml, was sequenced using the Illumina HiSeq 2500 platform to generate sequence data for further analysis.

### Genome assembly and completeness determination

Read quality was assessed using FastQC version v0.11.9. Paired-end reads were cleaned using Fastp (v0.19.1).^17^ Prinseq and NxTrim (v0.4.3) were used to clean the mate-pair reads.^18^ Nanopore reads were quality-checked with Nanoqc and Prinseq, and FMLRC was used to clean the Nanopore reads.^19^ The genome was assembled using Illumina mate-pair and pair-end reads along with error-corrected Nanopore reads using SPAdes v3.11 with a K-mer size ranging from 25-97 and a step size of 8.^20^ Reference-independent scaffolding was performed using BOSS.^21^ The draft assembly was evaluated using QUAST (v5.0.2),^22^ and completeness was evaluated using BUSCO v5.3.2.^23^ The presence of complete chromosomes was detected by identifying telomeres using the script available at https://github.com/JanaSperschneider/FindTelomeres. GenomeScope^24^ was used to find the uniqueness and heterozygosity of the genome. Genomes of *Apiospora pterosperma* (ASM1997648v1), *Apiospora rasikravindrae* (ASM2260543v1), and *Apiospora saccharicola* (ASM1900006v1), *Arthrinium phaeospermum* (ASM650353v1), *Arthrinium KUC21332* (ASM1716395v1), and *Arthrinium puccinioides* (ASM2241466v1) were re-analyzed and used for comparative genomics. We have also included *Nigrospora oryzae* (ASM1675884v1), a plant pathogen causing panicle branch rot disease in *Oryza sativa* (rice), as an outgroup.^25,26^

### Structural and functional annotation of the genome

Initial gene prediction was done with Augustus (V3.4.0),^27^ followed by providing Augustus predicted gene models and hints files from RNAseq to Funannotate (v1.6.0) for final gene prediction.^28^ Metabolic pathway prediction and functional annotation of the genomes were carried out using the KEGG-KAAS server (Supplementary Figure S1C)^29^ and eggNOG-mapper v2.1.9 (http://eggnog-mapper.embl.de/).^30^ Annotation files from eggnog (Supplementary Table S1)^31^ were plotted using the Seaborn heatmap package implemented in Python. Gene number difference between the species was calculated using Fisher’s exact test.

### Prediction of Carbohydrate-Active EnZyme families

The prediction of Carbohydrate-Active EnZyme (CAZymes) was carried out using the dbCAN3 web server (dbCAN3 server (unl.edu))^32^ against the CAZy database as a reference (http://www.cazy.org/). The CAZymes with prediction threshold (*E*-value < 1e^−10^) were classified as Glycoside Hydrolases (GHs), Glycosyl Transferases (GTs), Carbohydrate Esterases (CEs), Polysaccharide Lyases (PLs), Auxiliary Activities (AAs), and Carbohydrate-Binding Modules (CBMs).^33^

### Gene enrichment analysis

Enrichment analysis of Gene Ontology (GO) from eggNOG files was conducted using the R package—clusterProfiler.^34,35^ Initially, gene IDs were mapped to GO terms using the OrgDb package of *Saccharomyces cerevisiae* (org.Sc.sgd.db) in Bioconductor. Subsequently, the enrichGO function assessed the significance of each enriched GO term within the input gene set, employing the Benjamini-Hochberg (BH) multiple testing correction at a significance threshold of 0.05.

### Inoculation of *A. malaysiana* into dicot and monocot plants

Genes implicated in endophytism in *Rhodotorula mucilaginosa* JGTA S1^36^ were used as a reference for identifying endophytic genes in *A. malaysiana* using BLASTP 2.13.0+.^37^ Moong and rice seedlings were sterilized with 2% NaOCl for 10 minutes and placed into sterile bottles containing filter papers moistened with MSO medium. The seeds were incubated in the dark to facilitate germination. Roots of 4- to 5-day-old seedlings were inoculated under sterile conditions with four-day-old fungal mycelia. The inoculated seedlings were transferred to tall test tubes containing solidified MSO medium and grown under a 16/8-hour light-dark photoperiod for two weeks. Root and shoot lengths were measured to evaluate the growth benefits provided by the fungus. Sectioning and staining of the root and shoot were done using lactophenol cotton blue for microscopic investigation.

### Evolutionary relationship between the members of Apiosporaceae

Whole genome Mash distances between the members of Apiosporaceae were computed using Mashtree (v1.2.0) with 1000 bootstrap iterations.^38^ OrthoFinder (v2.5.4)^39^ was used to identify orthologous groups and construct a rooted species tree. Shared or unique orthologous genes were identified using the web-based tool OrthoVenn3.^40^ CAFE5 from OrthoVenn3 was used to analyze the expansion and contraction of gene clusters by evolution.^41^

### Prediction of effectors

The predicted protein sequences were first checked to determine whether or not there is a signal peptide with SignalP (v5.0b).^42^ The secretory proteins lack a transmembrane region (with TMHMM (v2.0)).^43^ Transmembrane proteins were discarded if they had a mitochondrial transit peptide (mTP) or chloroplast transit peptide (cTP) signal, as predicted with TargetP-2.0.^44^ Effector prediction was carried out using EffectorP (v3.0).^45^

### Speed genome prediction

Intergenic distances between the genes were calculated using an in-house script from gff3 files as described by Mandel et al.^46^ The 5′ and 3′ flanking intergenic regions (FIRs) were grouped with bin breaks for all the genes. The distribution of genes with effectors and CAZymes was plotted as a contour plot using ggplot2 in R version 4.2.2.^47^ Average FIRs of core genes, effectors, and CAZymes were plotted as boxplots using Python’s Matplotlib pyplot module.^48^

### Prediction of simple sequence repeats (SSRs) in the genome

Simple sequence repeat (SSRs) motifs with 2-10 bp length and repeated at least 5 times were detected using GMATA on both strands.^49^ GC percentage, SSR density (number of bases covered by SSRs/genome size in MB), SSR coverage (% of genome covered by SSRs), and SSR frequencies and motif abundances were computed using in-house Python scripts. (https://github.com/computational-genomics-lab/scripts-for-SSR-project). All the dimer, trimer, and tetramer SSRs were plotted using the seaborn package of Python.^50^

### Differential expression of genes in the presence of YPD and cellulose-only media

Fungal cultures were grown in YPD media (control condition) and 2% cellulose-only media (treated condition) in the dark at 28 °C, 150 rpm for 50 hours. RNA was isolated using the Trizol (Invitrogen) method and was quality-checked with a Qubit fluorometer (Thermo Scientific).

The RNA samples were processed for library preparation and sequenced using Illumina MiSeq technology. The quality of raw reads was checked using FastQC, followed by adapter trimming using Bbduck. High-quality reads were aligned to the genome with three different methods, e.g. STAR (v2.7.8a),^51^ HISAT2 (v2.2.1),^52^ and BOWTIE2 (v2.4.2).^53^ Samtools (v1.11)^54^ and featureCounts (v2.0.1)^55^ were used to count the number of reads aligned against a genomic feature. Differential expression analysis was done using deseq2.^56^ Transcripts were assembled using Trinity (v2.2.0),^57^ followed by quantification with Kallisto (v0.46.2).^58^ Count files were merged for expression analysis using the Degust online web-based program (https://degust.erc.monash.edu/).^59^ All expressed transcripts were filtered with a *P* value < 0.05, log_2_ fold expression change of 2 with false discovery rate (FDR) < 0.1. Assembled transcripts were mapped to the gene models using BLAT.^60^ Differentially expressed genes were visualized with Integrated Genomics Viewer (IGV) (Supplementary **Fig. S6**).^61^

### Cloning of cellulase genes

Two sets of upregulated genes in response to cellulose were selected for cloning and expression in bacterial system: Set 1 (APM_011205—gene 1, APM_012467 - gene 2, APM_002619 - gene 3) and Set 2 (APM_000720—gene A, APM_013033 - gene B, APM_009931 - gene C), each with distinct physicochemical properties (Supplementary **Table S2**). Genes 1, 2, and 3 were cloned into the pET-30a(+) vector, while genes A, B, and C were cloned into pET-22b(+). The genes and pET-22b(+) vector were restriction digested with NEB Restriction Endonucleases (REs) at 37 °C for 1 hour. Ligations were performed using Promega T4 DNA Ligase with a 3:1 molar ratio of gel-purified insert DNA, and constructs were transformed into DH5α cells via heat shock (42 °C, 45 sec). 10 to 20 colonies indicated high ligation efficiency. Double digestion confirmed successful cloning, with bands matching the expected sizes for gene B (~1029 bp, Fig. 9A) and gene C (~1230 bp, Fig. 9B).

**Fig. 1.**
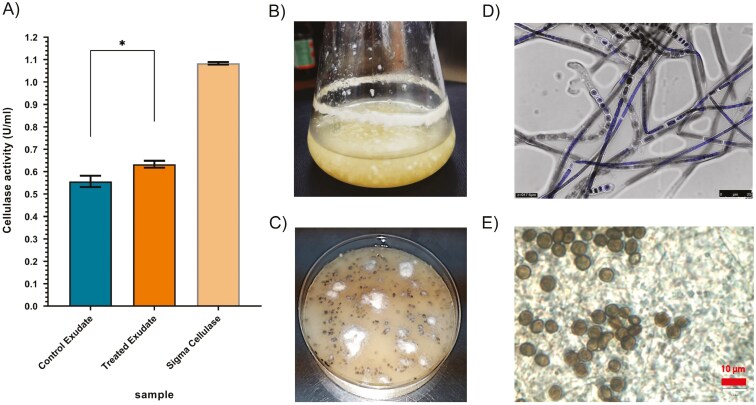
Cellulolytic activity of the exudates and microscopy of *A. malaysiana.* A) Cellulase activity of exudates grown in normal YPD media and 2% cellulose media in *A. malaysiana*, compared with sigma cellulase (used as positive control). B) 72-hour old liquid broth culture in YPD media. C) Cultures grown in YPD agar plate for three weeks. D) Confocal microscopy of DAPI stained mycelia. E) Microscopic image of conidia at 100X magnification showing lenticular side view and globular frontal view with a distinct equatorial slit.

**Fig. 2.**
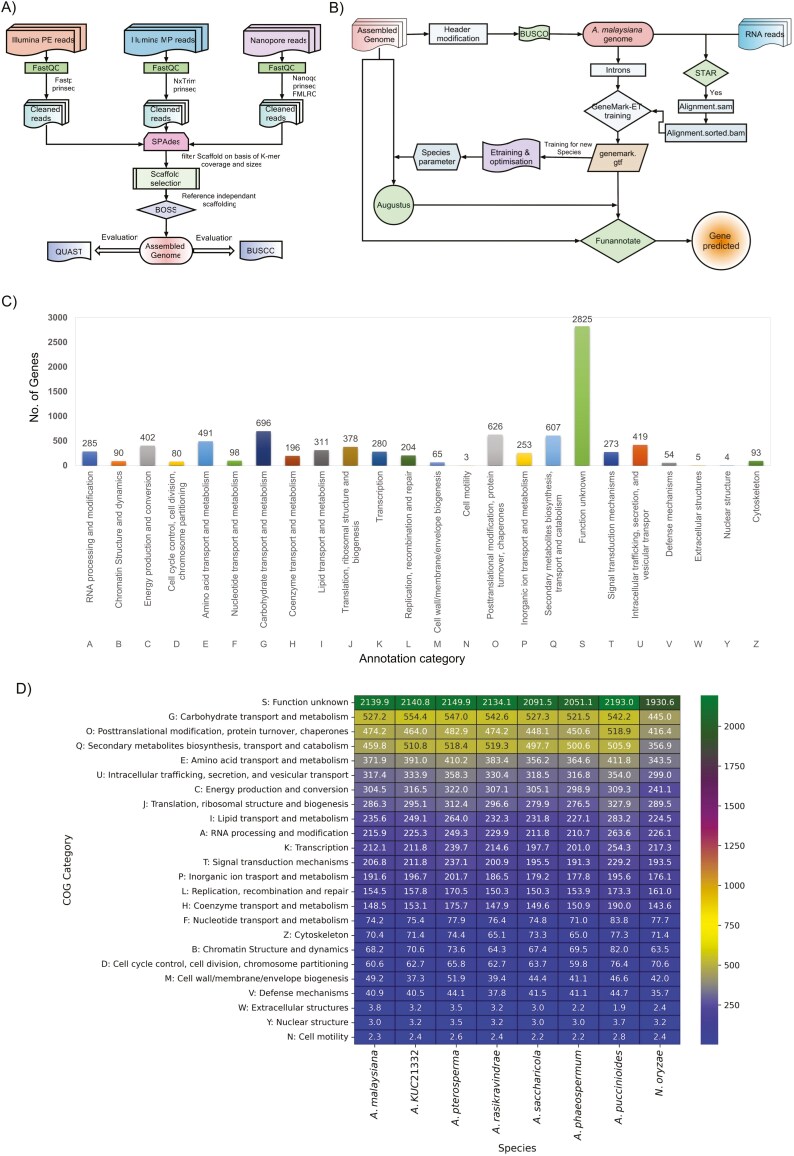
Annotation and assembly protocol followed in analyzing the genome of *A. malaysiana.* A) Schematic representation of genome assembly protocol followed using a combination of paired end, mate pair and Oxford Nanopore long reads. B) Training of gene models and gene prediction workflow for genome annotation in *A. malaysiana* with Augustus and Funannotate. C) Functional gene annotation of *A. malaysiana* shown in a category wise manner: X -axis shows function category and Y axis shows the number of genes. D) Heatmap of COG categories of genes normalized per 10000 genes in all the 8 species studied.

**Fig. 3.**
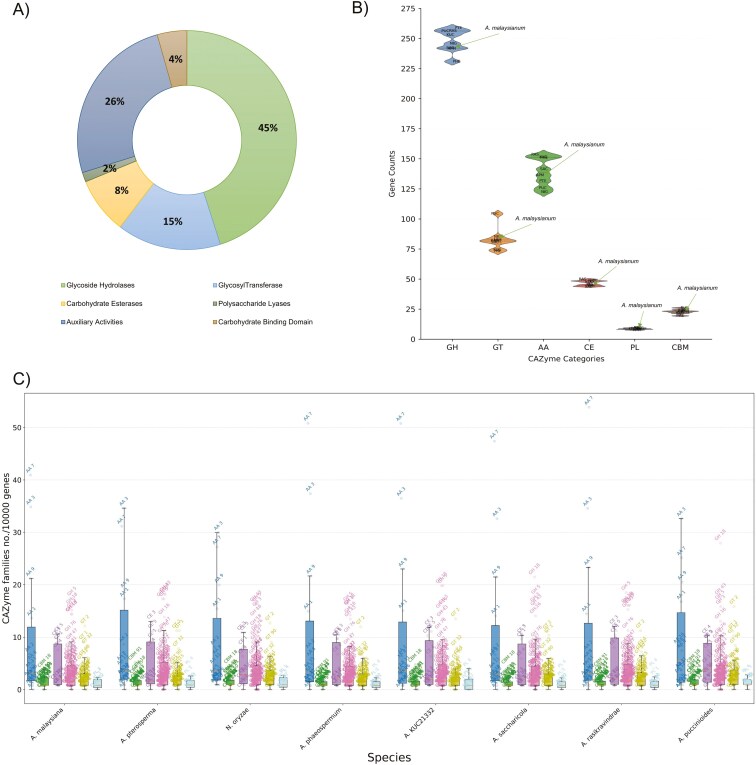
CAZyme profile of all the genomes studied. A) CAZyme categories of *A. malaysiana.* in percentage depicting GH class as the most abundant of all. B) CAZyme categories in all the genomes studied indicate a predominance of GH classes across all the species. C) Members of CAZymes families plotted in a box plot showing their relative number after normalizing the values per 10000 genes.

**Fig. 4.**
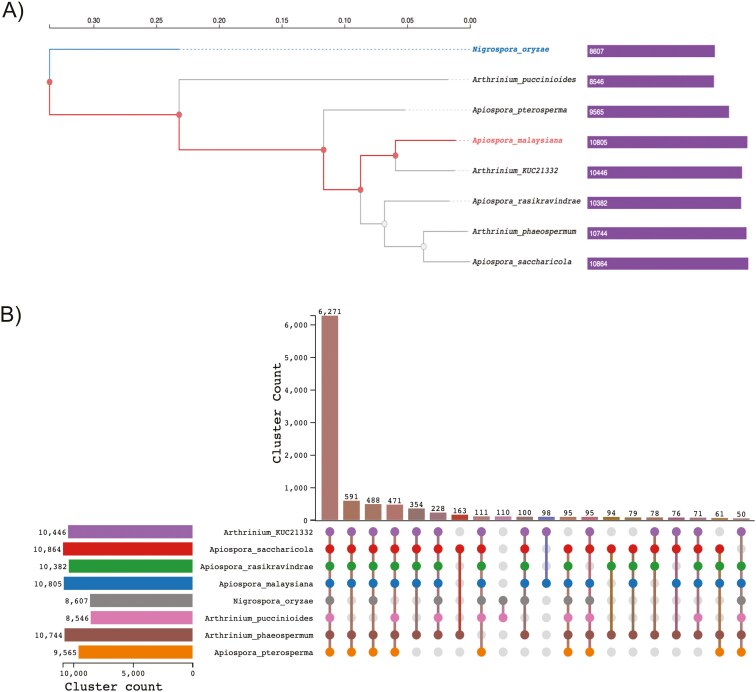
Evolutionary relationship among the species. A) Genome tree construction using FastTree method using the gene ortholog clusters implementing Maximum likelihood method. B) Upset-JS plot illustrating the distribution of homologous genes shared among the studied species. The largest cluster apart from the common genes are shared between endophytes (591) and there is a distinct cluster unique to the two parasites under study (110).

**Fig. 5.**
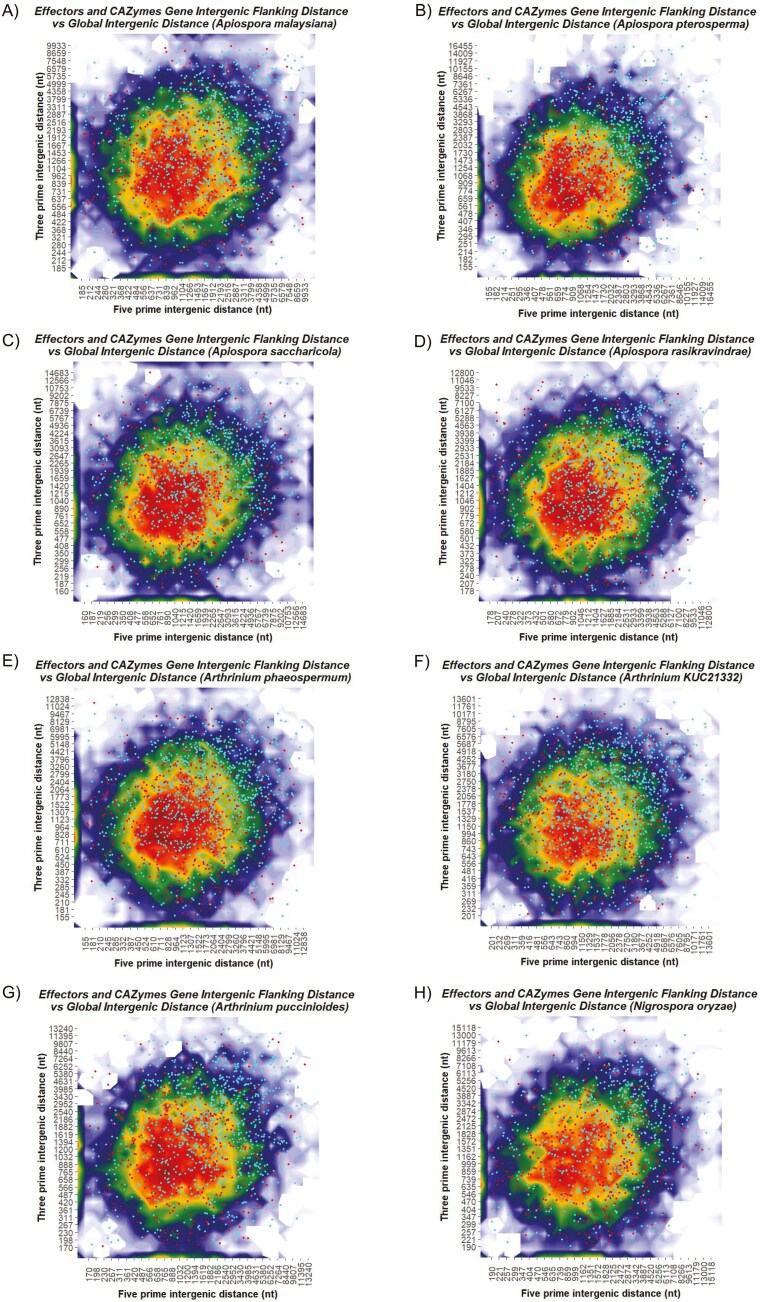
Two-dimensional contour plot showing two-speed genome architecture of the species under study. The color gradient represents gene density, with lighter colors indicating less dense or gene-sparse regions, whereas darker colour represents regions with densely packed genes in the genome. Cyan dots represent the effectors, and red dots represent CAZymes. Distinct localization of Cyano dots on the top right panel indicates effectors are localized more towards the gene sparse regions indicating a bi-partite genome architecture.

**Fig. 6.**
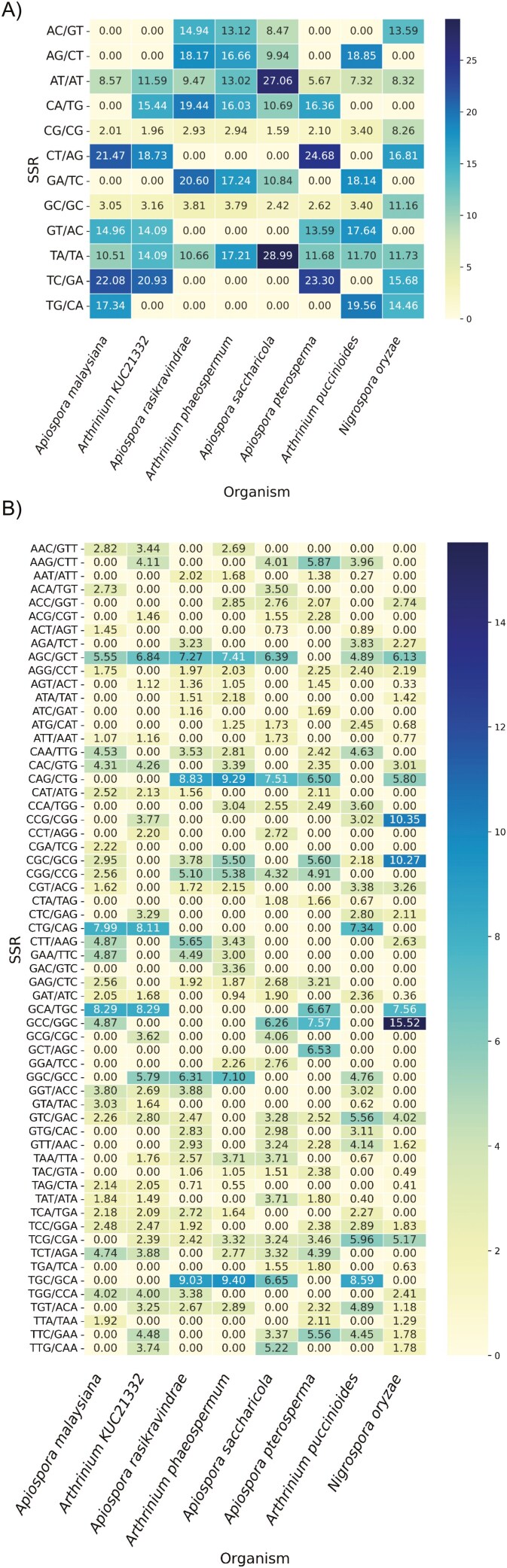

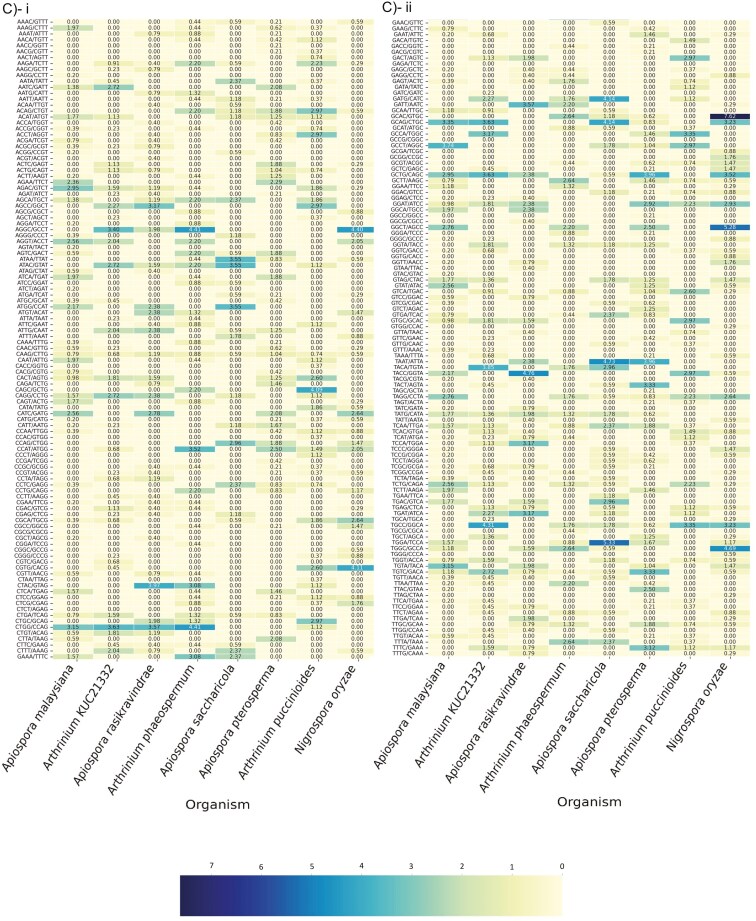
Heatmap of simple sequence repeats (SSRs) across all the species. (A) Dimer motifs, (B) Trimer motifs, and (C) Tetramer motifs in the group of organisms represented in two columns. The values represented in the cells are normalized as number of motifs per Mb of genome.

**Fig. 7.**
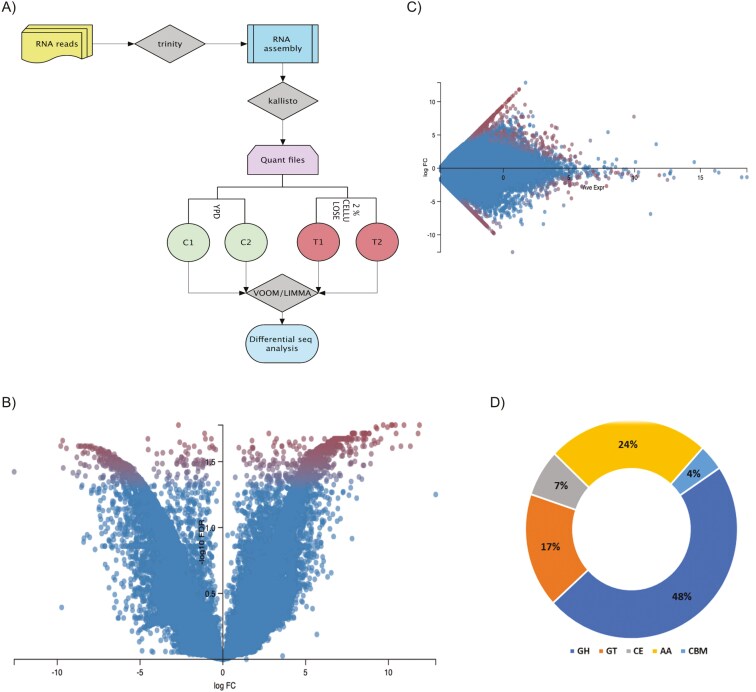
RNA-Seq analysis. A) RNA-Seq analysis workflow. B) Volcano plot of expressed transcripts, red dots represent highly expressed and blue dots are less expressed transcripts, with log fold change on X—axis and -log_10_ false discovery rate on Y- axis. C) MA plot of expressed transcripts, with average expression at X—axis and log fold change at Y -axis. D) CAZymes categories or families upregulated in RNA-Seq analysis in percentage (GH: Glycoside hydrolase; GT: Glycosyl transferase; CE: Carbohydrate esterase; CBM: Carbohydrate-binding module; AA: Auxiliary activities).

**Fig. 8.**
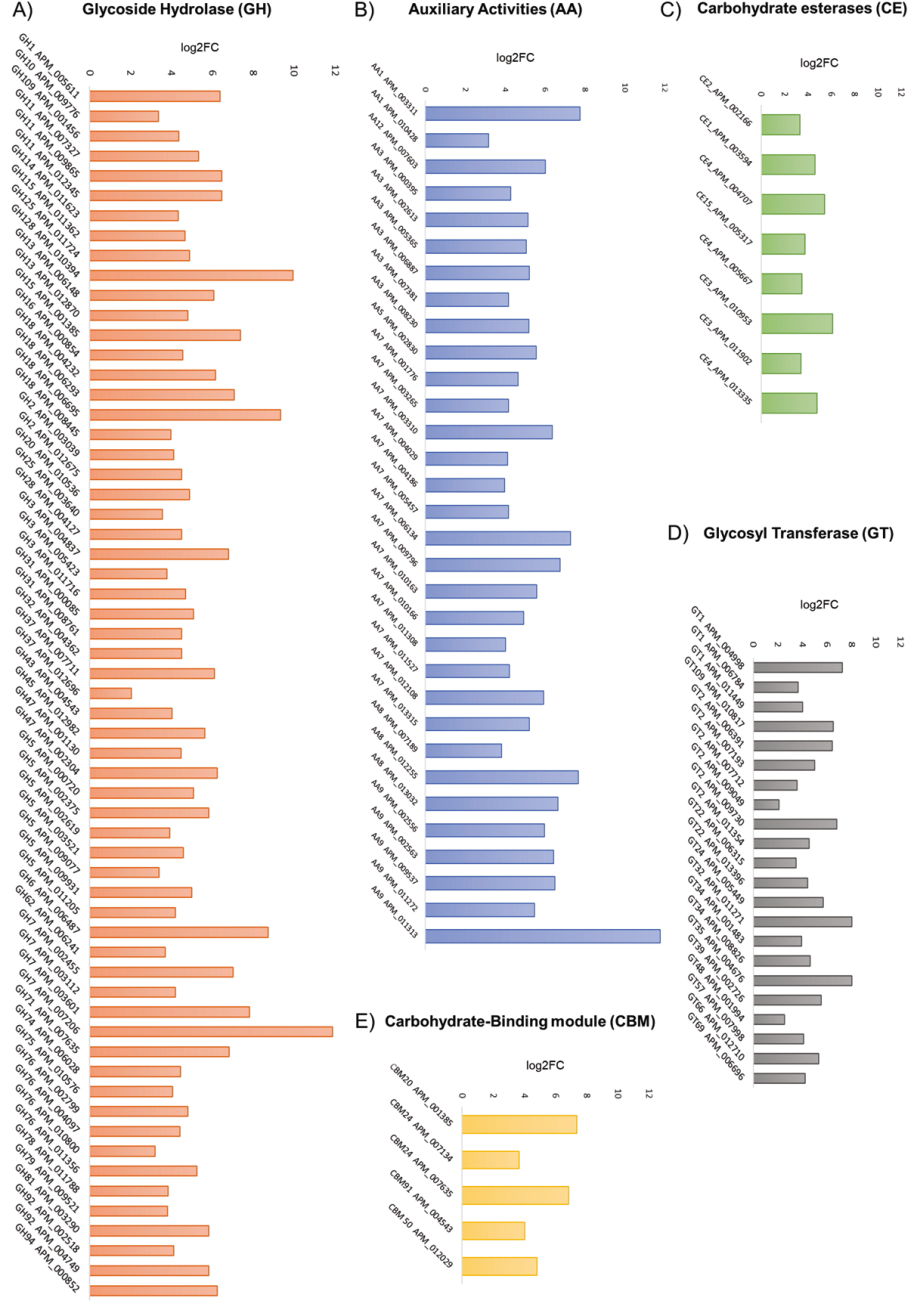
Upregulated CAZymes with their family and their expression profiles in the transcriptomic study of *A. malaysiana*. The expression levels of each CAZyme are represented in log₂ fold change on Y—axis and CAZymes gene ids at X—axis. (A) Glycoside hydrolases, (B) Auxiliary activities, (C) Carbohydrate esterases, (D) Glycosyl transferases, and (E) Carbohydrate-binding modules.

**Fig. 9.**
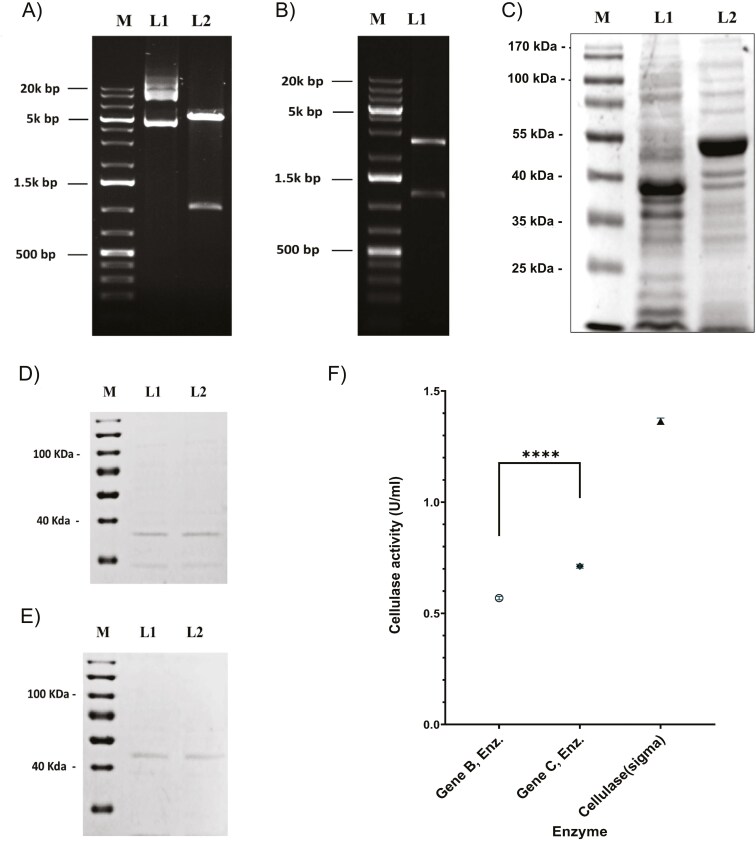
Recombinant protein expression and activity analysis. A) Double digestion of cloned cellulase gene B (*APM_013023*) (M: DNA marker, L1: Undigested plasmid, L2: Digested plasmid). B) Double digestion of cloned cellulase gene C (*APM_009931*) (M: DNA marker, L1: Digested plasmid). C) Expression of recombinant protein with 0.5 mM IPTG (M: Protein marker, L1: Gene B, L2: Gene C). Purified protein of D) gene B, and E) gene C (L1: 250 mM, L2: 500 mM imidazole-eluted fractions). F) Enzyme activity of genes B and C recombinant enzymes, recombinant enzymes are present on X—axis, whereas their activity is represented in Unit/ml in one minute on Y—axis.

### Expression and purification of cellulase genes

The verified constructs were transformed into BL21 DE3 competent cells for protein expression. After overnight growth, a single colony was inoculated into an LB medium with antibiotics. Set 1 genes were cultured in LB with kanamycin (50 μg/mL) and induced with IPTG at 16 °C for 14 hours **(Supplementary Fig. S8A**-**C**), while Set 2 genes were cultured with 0.5 mM IPTG and ampicillin (100 μg/mL) under similar conditions. Protein expression was confirmed via 12% SDS-PAGE analysis of total lysates (Fig. 9C).

Cells were harvested and lysed for large-scale purification in a buffer containing 50 mM Tris-HCl (pH 8.0), 300 mM NaCl, and 0.1% Triton X-100. The pellet was solubilized with 2M urea and sonication (15s on, 45s off) for 15–20 minutes of enhanced cell lysis. The lysate was filtered using Amicon Ultra centrifugal filters (MWCO 10 kDa) to remove urea and contaminants. Protein purification was performed using Ni-NTA affinity chromatography. Nonspecific proteins were washed with 20 mM and 50 mM imidazole, and histidine-tagged proteins were eluted with higher imidazole concentrations (200 and 500 mM), disrupting histidine-metal interactions (**Fig. 9D and 9E**).

The Bradford assay, with a BSA standard curve (100-1200 mg/mL), was used to quantify protein concentration. CMCase activity was measured using CMC as substrate. The released glucose, a product of the enzyme’s activity, was quantified by the DNS assay at 540 nm following Ghose’s protocol.^15,62^ The amount of liberated glucose was determined by comparing it with the glucose standard curve and marketed cellulase as a positive control (Sigma cellulase). Enzyme activity was found utilizing the formula:


$$\begin{array}{lll} \; & Enzyme\;activity\;\left(U\right)=\;\;\;\; & \frac{Micromole\;of\;glucose\;released}{Reaction\;time\left(min\right)\times Volume\;of\;enzyme\;used\left(ml\right)} \end{array}$$

One U (μmol/min) of enzyme activity is defined as the amount of enzyme that catalyze 1 µmol of substrate per minute under a specific assay conditions.^63^

## Results and discussion

### Exudate of *A. malaysiana* has carbohydrate hydrolyzing property

The exudates of the fungus grown in standard and cellulose-only media were analyzed for carbohydrate-degrading activities. A crude CMC assay conducted after concentrating the exudate 500-fold—revealed significant activity of 0.55 ± 0.0206 U/ml when the fungus was grown in YPD. This activity increased to 0.63 ± 0.0124 U/ml when the fungus was grown in a 2% cellulose-only medium (Fig. 1A).

### Fungal strain was identified as *A. malaysiana*

The fungus exhibits distinct morphological features in both submerged and solid cultures. In submerged culture, it forms visible clumps or cloud-like aggregates of hyphae, or spherical balls with thin filamentous hair-like projections (Fig. 1B). On solid plates, it appears as black, cloudy spots (Fig. 1C). Microscopic examination reveals dense, branched septate hyphae with a diameter of 2-3 μm. Confocal microscopy, following DAPI staining, shows multinucleated hyphal structures (Fig. 1D). The spores or conidia are nearly spherical when viewed from the front and lenticular from the side, measuring 5-7 μm in diameter on the surface and 3-4 μm from the side—a characteristic feature of *Apiospora* species.^9^ The conidiogenous cells are clustered together (Fig. 1E). Amplification of the ITS region using specific primers yielded a 630-nucleotide amplicon (**Supplementary Fig. S1A**). Sanger sequencing of this ITS region confirmed over 97% similarity to *A. malaysiana*.

### The genome of *A. malaysiana* shows low heterozygosity

The genome was assembled using 44,370,409 × 2 reads of 150 bp length, comprising 38,509,379 × 2 paired-end reads, and 5,861,030 × 2 mate-pair reads, approximately at 230× coverage. Additionally, 29,721 error-corrected nanopore reads were used in assembly, resulting in 91 contigs that were scaffolded into 43 scaffolds using BOSS (Fig. 2A). The final genome (46Mb) is nearly complete (99.1% BUSCO completeness against sordariomycetes_odb10 database), as is evident from the GenomeScope 2.0 output using K-mers approach (Supplementary Fig. S1B) (**Table 1**).

**Table 1.** Assembly and feature statistics, genome completeness using sordariomycetes_odb10 for all the 8 species.

Organism	Total length	Scaffold/ Contig N50	Scaffold /contigs No.	Completeness %	Funnannotate predicted	Gene length	GC %	Gene length/Genome
*Apiospora malaysiana*	46,686,835	2,429,633	43	98.4	13,200	1,967,2515	52.54	42.14%
*Apiospora pterosperma*	45,078,112	4,729,002	14	96.4	11,553	17,768,973	51.63	39.42%
*Apiospora saccharicola*	55,763,147	3,269,722	37	98.3	13,501	19,964,342	50.08	35.8%
*Apiospora rasikravindrae*	46,448,847	2,356,432	32	98.4	12,440	18,886,937	52.67	40.66%
*Arthrinium phaeospermum*	49,055,814	3,733,262	19	94.0	13,383	20,026,483	53.05	40.82%
*Arthrinium KUC21332*	49,349,353	3,570,796	50	98.0	12,606	19,307,521	52.09	39.12%
*Arthrinium puccinioides*	40,146,223	6,167,320	10	95.1	10,734	17,090,548	53.33	42.57%
*Nigrospora oryzae*	43,754,591	4,037,616	15	98.0	11,022	17,167,761	58.19	39.24%

Telomeres were observed at both ends of several scaffolds in *A. pterosperma* (apio_9 and apio_11) and *A. saccharicola* (apio_1), suggesting complete chromosome-level assemblies for these chromosomes. However, in all organisms analyzed, including *A. malaysiana*, telomeres were only present on one end of most scaffolds (Supplementary **Table S3****).** The genome of *A. malaysiana* exhibited low levels of heterozygosity (10.8%), indicating a predominantly clonal population that reproduces vegetatively.^64^ The absence of a well-defined sexual morph in lab-grown cultures, even if it has an intact MAT locus (Supplementary **Fig. S1D**).

### A higher number of genes were predicted using Funannotate

Using Augustus, 12,056 gene models were predicted in *A. malaysiana*. However, the annotation with Funannotate resulted in a 9% higher number of genes (13,200) in *A. malaysiana* (Fig. 2B). In all eight species under study, the predicted genes ranged from 10,734 to 13,501, indicating that 35% to 42% of the whole genome was coding. As expected, the obligatory pathogens, *A. puccinioides*, and *N. oryzae,* had the fewest genes, a characteristic of pathogenic organisms.^65^

### Occurrence of specific carbohydrate degrading enzymes in the ascomycetes species re-iterates their lifestyle preferences

Across the 8 fungal species studied, a significant fraction of genes had unknown functions. In *A. malaysiana*, abundant protein classes include those involved in carbohydrate metabolism (527.22/10000 gene), post-translational modifications, protein turnover, chaperones (474.19/10000 gene), and secondary metabolite biosynthesis, transport, and catabolism (459.80/10000 gene) (Fig. 2C, D). In contrast, in pathogen *N. oryzae,* a reduced number of secondary metabolite biosynthesis, transport, and catabolism genes were found after analyzing using Fisher’s exact test (Supplementary Table S1 sheet no.10). This reduction reflects its specialized lifestyle adaptation.^66^ In all the species, glycoside hydrolases (GH) dominate the CAZyme repertoire, with the most abundant families being GH3, GH5, GH16, GH18, and GH43, involved in cellulase, hemicellulase, and pectinase activities (Fig. 3A–C) (Supplementary **Fig. S2A****) (**Supplementary **Table S4****and****S5**).^67,68^ Glycosyltransferases (GTs), including GT1, GT2, and GT32 families, facilitate glycosylation of natural products (Supplementary **Fig. S2B**).^69^ Auxiliary Activities (AA), also known as gluco-oligosaccharide oxidases (GOO), primarily AA7, participate in lignin detoxification and lignocellulose breakdown (Supplementary **Fig. S2C**).^70,71^ Carbohydrate esterases (CEs), such as CE5 (cutinases), are abundant in *A. malaysiana* and play roles in plant-pathogen interactions (Supplementary **Fig. S2D**).^72,73^ Polysaccharide lyases (PLs) specifically target polysaccharide chains in uronic acid and the. PL4 class, degrade rhamnogalacturonan^74^ (Supplementary **Fig. S2E**). The two pathogen species and *A. malaysiana* have slightly reduced number of PL4 class, possibly indicating monocots as host.^75^ The carbohydrate binding module (CBM) binds with the substrate and influences the overall enzymatic activity.^76^*A. malaysiana* contains five CBM18 genes associated with chitinase activity, potentially protecting fungal cell walls against hydrolysis^77^ (Supplementary Fig. S2F).

### *A. malaysiana* acts as a necrotroph in rice plants

Homologs of several plant hormone genes, such as auxin biosynthesis, cytokinin metabolism, abscisic acid biosynthesis, gibberellin biosynthesis, and sterol biogenesis, were found that were similar to plant hormone genes of *R. mucilaginosa* JGTA-S1^36^ (**Table 2**). However, no significant beneficial growth effect was noticed with moong seedlings, although the plant hormone genes were seen to be transcriptionally active (Supplementary **Fig. S3A**). However, upon inoculation to the Rice plant, a definite necrotrophism was noticed in the host, and the root sections showed fungal mycelium through the root cells (Supplementary **Fig. S3C****-****D**). The prominent absence of haustoria or any other distinctive structure prevalent in biotrophism was missing.^78–80^ On the contrary, the inoculated roots of Moong plants showed no fungal mycelium, possibly indicating Moong to be a non-host (Supplementary **Fig. S3B**).

**Table 2.** Differentially expressed plant phytohormone genes during cellulose treatment.

Hormone	Query gene ID	Query gene name	Gene ID in *A. malaysiana*	Log_2_ expression
**Auxin biosynthesis**	g674.t1	Monoamine oxidase	APM_005715	5.35826081
	g1891.t1	Aromatic-l-amino-acid decarboxylase	APM_003807	2.358731871
**Cytokinin metabolism**	g986.t1	Cytochrome P450, family 3, subfamily A	APM_002968	2.508032269
	g2843.t1	putative 12-oxophytodienoate reductase 11	APM_004110	−2.058596557
	g4607.t1	4-coumarate-CoA ligase	APM_012959	-3.175843756
**Sterol biogenesis and metabolism**	g268.t1	C-5 sterol desaturase	APM_007979	−1.611732407
	g309.t1	C-3 sterol dehydrogenase	APM_002291	−1.499466354
	g670.t1	C-22 sterol desaturase	APM_012157	−1.701006648
	g1695.t1	Oxysterol-binding protein	APM_003146	1.165689746
	g2120.t1	C-3 sterol dehydrogenase	APM_008378	0.7416372
	g2335.t1	Delta24(24(1))-sterol reductase	APM_001066	1.142421902
	g2391.t1	C-8 sterol isomerase (Erg-1)	APM_008534	−1.040877838
	g2848.t1	Oxysterol-binding protein	APM_000563	1.57093796
	g3135.t1	Oxysterol-binding protein	APM_004301	0.436766293
	g3289.t1	Oxysterol binding protein	APM_010518	2.775006064
	g3596.t1	Oxysterol-binding protein	APM_000846	0.485833087
	g4048.t1	C-4 methyl sterol oxidase	APM_004594	−5.852043369
	g4665.t1	Sterol O-acyltransferase	APM_013043	3.890646534
	g4804.t1	Sterol 24-C-methyltransferase	APM_002694	−1.058963947
	g5096.t1	Sterol 3-beta-glucosyltransferase (Autophagy-related protein 26)	APM_007230	2.967285739

### Pathogenic species and endophytes have specific ortholog gene clusters representing their pathogenicity and endophytic adaptations

Genome tree construction using OrthoFinder indicates that *A. malaysiana* is closely related to *A. KUC21332* isolate, with *Nigrospora oryzae* positioned as an outgroup (Fig. 4A). Analysis using the CAFÉ tool suggests that *N. oryzae* and *A. puccinioides* diverged from the other species approximately 1.5 million years ago. There were three unique orthologs found between pairs of species, e.g. *N. oryzae* and *A. puccinioides*; *A. malaysiana* and *A. KUC21332*; and *A. phaeospermum* and *A. saccharicola*, of which the latter group formed the largest cluster pair (163 pairs). *A. puccinioides* exhibited 152 expanded and 464 contracted gene clusters, while *N. oryzae* showed 141 expansions and 431 contractions. These gene contraction patterns are characteristic of obligatory pathogens, reflecting genome reduction to adapt to a host-dependent lifestyle (**Supplementary Fig. S4A**).^81,82^

Ortholog analysis across all organisms identified 6,271 shared clusters comprising 58,754 proteins (Fig. 4B). The most enriched biological pathway was mRNA transport, with helicase and GTPase activities as the dominant molecular functions (**Supplementary Fig. S5A****-****H**). Across all organisms, 12,462 clusters were identified, including 4913 single-copy clusters representing 98,439 proteins. Singletons accounted for 3439 clusters (3.49%) (**Table 3**). A total of 591 clusters (4377 proteins) were shared among endophytic *Apiospora* and *Arthrinium* species, excluding pathogens (Supplementary **Fig. S4B**). Most prominently, the cluster codes for genes responsible for secondary metabolite biosynthesis, nitrogen compound metabolic process, response to jasmonic acid and salicylic acid, seed germination, and antibiotic biosynthetic process.^83^

**Table 3.** Protein cluster statistics as determined by OrthoVenn3.

Organisms	No. of proteins	No. of clusters	No. of singletons
*Apiospora saccharicola*	13,501	10,864	334
*Arthrinium phaeospermum*	13,383	10,744	345
*Apiospora malaysiana*	13,200	10,805	370
*Arthrinium KUC21332*	12,606	10,446	240
*Apiospora rasikravindrae*	12,440	10,382	245
*Apiospora pterosperma*	11,553	9565	299
*Nigrospora oryzae*	11,022	8607	898
*Arthrinium puccinioides*	10,734	8546	708

Between the two pathogenic species, *A. puccinioides* and *N. oryzae*, 110 unique clusters with 264 genes were identified (**Supplementary Fig. S4C**). Key processes specific to parasites included sporulation, pectin and mannan catabolism, protein mannosylation, quinate metabolism, pathogenesis, and vesicle-mediated transport. The largest shared cluster (664, with 14 proteins) is related to sporulation (Swiss-Prot Hit—A0A0B5L7R4) (Supplementary **Table S2**). Sporulation is a process in which pathogens dispense spores for germination and infection on new hosts and is often associated with pathogenesis.^84,85^ The presence of exclusive sporulation clusters in pathogenic species could shed light on their specific lifestyle.

### Position and spacing of effectors indicate genome compartmentalization in Apiospora

A. *malaysiana* stands out within its group for potentially having a large arsenal of 1,530 effector proteins, with 995 unique effectors in the group, indicating it may have evolved a more extensive repertoire to aid in effective host plant colonization^86^ (**Table 4**).

**Table 4.** No. of predicted effectors in all the 8 species

Organism	Signal peptides	Tmhmm	Target P	Effectors
*Apiospora malaysiana*	1492	204	4	1530
*Apiospora pterosperma*	1303	189	4	1267
*Apiospora saccharicola*	1540	233	3	1537
*Apiospora rasikravindrae*	1505	219	6	1542
*Arthrinium phaeospermum*	1534	218	2	1516
*Arthrinium KUC21332*	1534	237	2	1619
*Arthrinium puccinioides*	1255	197	2	1235
*Nigrospora oryzae*	1207	177	2	934

We computed the genes’ three-prime and five-prime intergenic distances in each species with predicted gene models (**Supplementary Table S6**). The distribution of effectors and CAZymes was plotted (Fig. 5). A ‘two-speed genome’ architecture was observed in filamentous pathogens, where TE-rich blocks containing effector genes were interspersed between regions with high gene density and low repeat content. These blocks showed high diversity and were prone to genomic rearrangements, supporting adaptation in the evolutionary race.^3,87^ However, fungi use the RIP mechanism to target repeats, protecting the genome from duplications and TE proliferation. Genomes with even TE dispersion and no compartmentalized structure are classified as ‘one-speed genomes’.^88^ The average FIR of all 5’ and 3’ were plotted and compared with FIRs of CAZYmes and effectors (Supplementary **Fig. S6**), in which we found that all *Apiospora* and *Arthrinium* species exhibited a two-speed genome architecture.

### Reduced SSR frequency is an indicator of lower potential for genetic variation in *A. malaysianum*

The number of simple sequence repeats (SSRs) in *A. malaysiana* was 7352, whereas it was the highest for *A. pterosperma*, for example, 8,390. This corresponds to a lower SSR coverage in *A. malaysiana* (0.29%) versus *A. puccinioides* (2.21%), suggesting reduced genetic variation in *A. malaysiana*, and low heterozygosity of this strain (section - The genome of *A. malaysiana* shows low hetrozygosity ). In contrast, the highest SSR coverage in obligatory pathogens indicates rapid adaptation and larger mutable DNA.^89^ Di-, tri-, and tetra-nucleotides comprise over 92% of SSRs, with TA/TA being the most common motif, while CA/TG motifs were absent in both pathogens and in *A. malaysiana* (Fig. 6) (Supplementary Table S7).

### Large clusters of secondary metabolites predicted from the genomes of *A. malaysiana* belonged to the NRPS class

Secondary metabolites were predicted using antiSMASH fungal version, that is, fungiSMASH of antiSMASH 7.0.^90^ A total of 63 secondary metabolite regions were identified in the genome of *A. malaysiana*. The majority of these are type 1 polyketide synthase (T1PKS), Non-Ribosomal Peptide Synthetase (NRPS), NRPS-like, Terpene, Fungal RiPP with POP or UstH peptidase types, and modification (fungal-RiPP), fungal-RiPP-like, etc. Only 8 of the 63 are highly similar (100 percent) to known clusters, implying that most are unknown and yet to be revealed. These metabolic clusters were localized in 25 scaffolds, while the rest of the scaffolds had none (**Supplementary Table S8**).

### Membrane trafficking, exosome, and CAZymes are upregulated during cellulose treatment

We have grown *A. malaysiana* in 2% cellulose-only media and compared the changes in gene expression with respect to YPD culture (Fig. 7**) (**Supplementary Table S9**).** The most upregulated genes were related to membrane trafficking (81), exosome (46), chromosome proteins (37), and transporters (28), along with cytoskeleton proteins, peptidases, inhibitors, and glycosyl transferases (Supplementary Table S10).

Intriguingly, in the absence of nutrition in the media with cellulose as the only substrate, several Phytohormone genes, especially the Auxin biosynthesis gene (**Table 2**) have been perturbed with a > -fold log_2_ upregulation. However, it is unclear whether the presence of cellulose in the media signals the presence of a potential host to establish an endophytic connection. Several CAZymes (19%) were upregulated, including GH, AA, CE, and CBM classes (**Fig. 8A–E**). Upregulation of CE4 classes (APM004707; APM005667; APM013335) is recorded in Cellulose only media (Fig. 8C). The upregulation of cell wall modification genes suggests that *A. malaysiana* adapts its cell wall to metabolize external cellulose.^91^

During host invasion, pathogens adapt to multiple strategies to evade host surveillance systems, including converting its Chitin to Chitolase with Chitin deacetylases of family CE4 and scavenging degraded Chitins with LysA domain proteins in CBM modules.^36^ There are several reports suggesting that fungal endophytism is an unstable strategy and the switch between endophytes to necrotrophic is much more common than thought earlier.^80^

### Purified protein had cellulolytic activities

The recombinant protein concentrations for genes B and C (from set 2) were determined by comparing absorbance to the BSA standard curve (Supplementary **Fig. S8D**). The glucose released from cellulose was quantified using the glucose standard curve (Supplementary Fig. S8E).^92^ Genes B and C were successfully expressed in the soluble fraction, while the genes from set 1 formed inclusion bodies. This may be due to their isoelectric point (pI); the gene from set 1 has a pI of 5, while genes B and C have pI values around 8. A study revealed that acidic proteins exhibit the highest average number of interactions. In contrast, basic proteins show the lowest number of interactions in both prokaryotic and eukaryotic proteomes,^93^ which may contribute to solubility loss.

Gene product B had 0.568 ± 0.006 U/ml/min activity, and gene product C had 0.711 ± 0.007 U/ml/min activity, with 1 U defined as the enzyme releasing 1 µmol of glucose per minute at pH 4.8, 50°C.^94^ Gene C showed slightly higher activity than gene B (Fig. 9F). Specific CMCase activity was 7.40 ± 0.08 U/mg for gene B and 8.08 ± 0.08 U/mg for gene C. These proteomic insights suggest *that A. malaysiana* holds significant potential for industrial enzymes and biofuel production applications.

## Supplementary Material

dsaf011_suppl_Supplementary_Figure_S1

dsaf011_suppl_Supplementary_Figure_S2

dsaf011_suppl_Supplementary_Figure_S3

dsaf011_suppl_Supplementary_Figure_S4

dsaf011_suppl_Supplementary_Figure_S5_A

dsaf011_suppl_Supplementary_Figure_S5_B

dsaf011_suppl_Supplementary_Figure_S6

dsaf011_suppl_Supplementary_Figure_S7

dsaf011_suppl_Supplementary_Figure_S8

dsaf011_suppl_Supplementary_Table_S1

dsaf011_suppl_Supplementary_Table_S2

dsaf011_suppl_Supplementary_Table_S3

dsaf011_suppl_Supplementary_Table_S4

dsaf011_suppl_Supplementary_Table_S5

dsaf011_suppl_Supplementary_Table_S6

dsaf011_suppl_Supplementary_Table_S7

dsaf011_suppl_Supplementary_Table_S8

dsaf011_suppl_Supplementary_Table_S9

dsaf011_suppl_Supplementary_Table_S10

dsaf011_suppl_Supplementary_Figures_S1-S8

## Data Availability

The raw reads, the assembly, and the annotation can be found at NCBI BioProject PRJNA484135 with the GenBank accession number GCA_006508115.1 for the assembly and the SRA accession number SRX5010283-84 for the raw reads. BioSample id is SAMN09760231. The strain was submitted to the Microbial Type Culture Collection and Gene Bank (MTCC) under accession number 13850. All the organism-related annotations are available at http://eumicrobedb.org:4000/.
