# Supplementary figures and images for: Comparative genomics of endophytic fungi Apiospora malaysiana with related ascomycetes indicates adaptation attuned to lifestyle choices with potential sustainable cellulolytic activity
Source: DNA Res. 2025 May 10;32(3):dsaf011. doi: 10.1093/dnares/dsaf011 (PMC12202052; doi:10.1093/dnares/dsaf011)

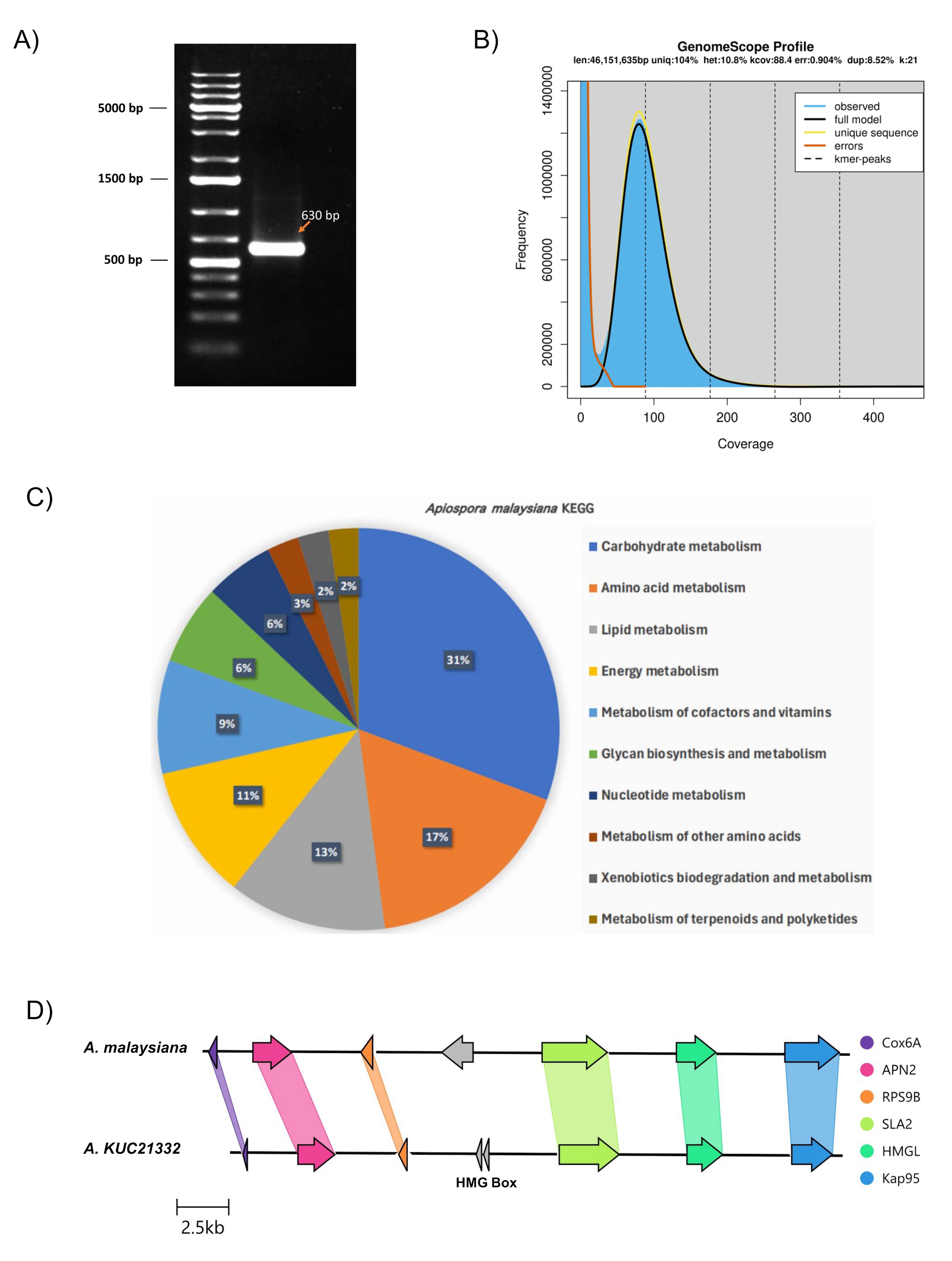

Supplement: dsaf011_suppl_Supplementary_Figure_S1 [file dsaf011_suppl_supplementary_figure_s1.jpeg]

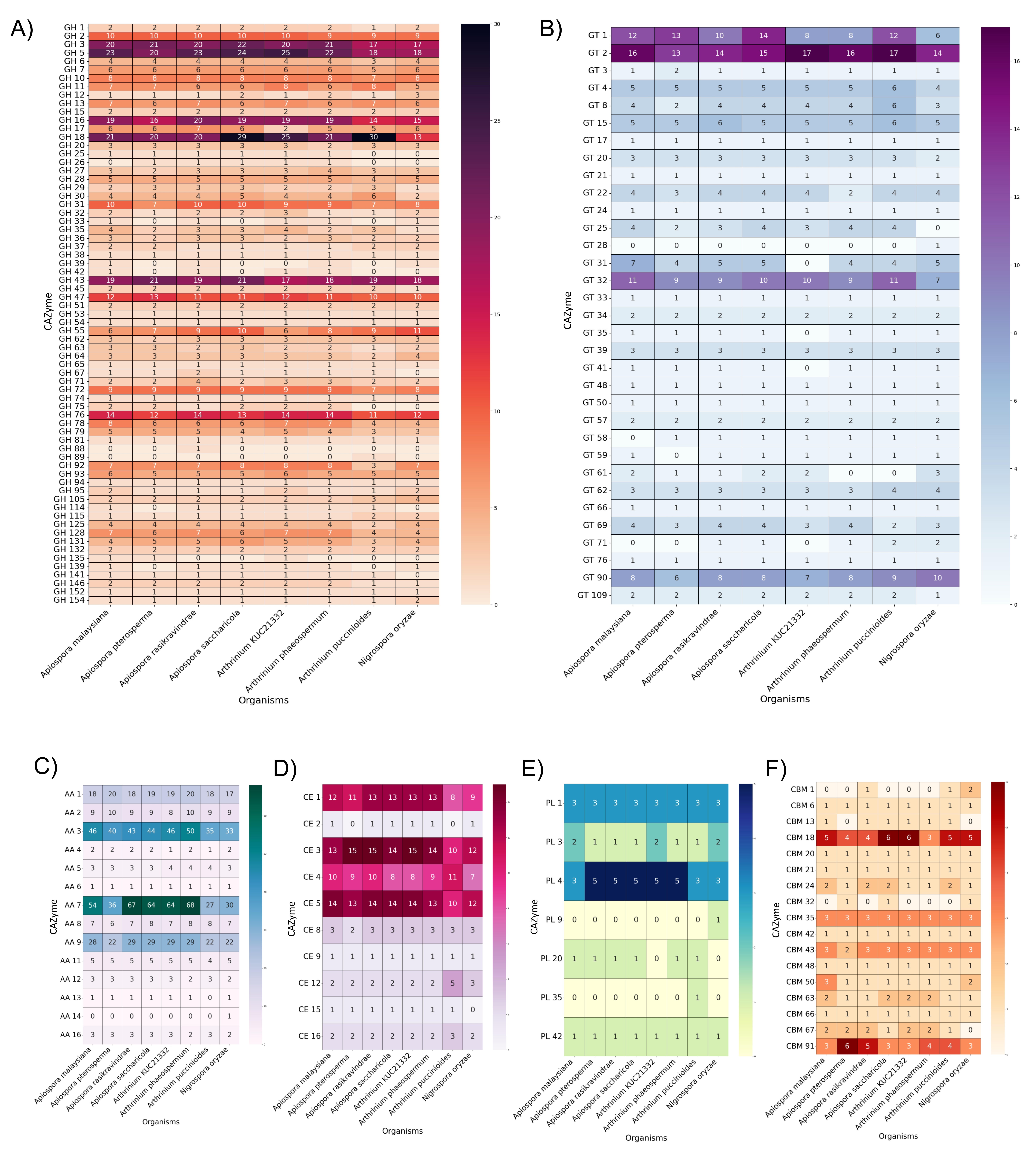

Supplement: dsaf011_suppl_Supplementary_Figure_S2 [file dsaf011_suppl_supplementary_figure_s2.jpeg]

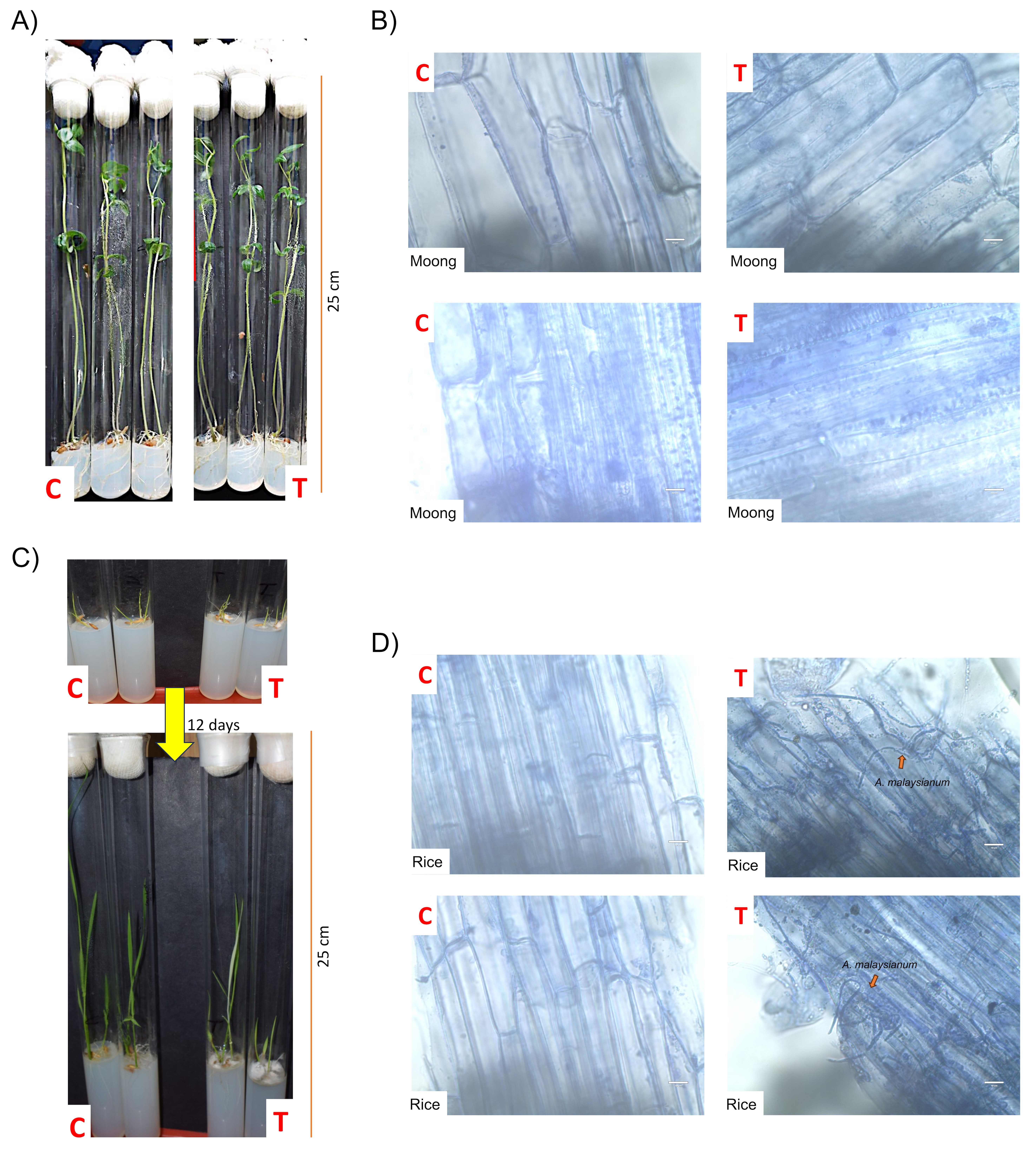

Supplement: dsaf011_suppl_Supplementary_Figure_S3 [file dsaf011_suppl_supplementary_figure_s3.jpeg]

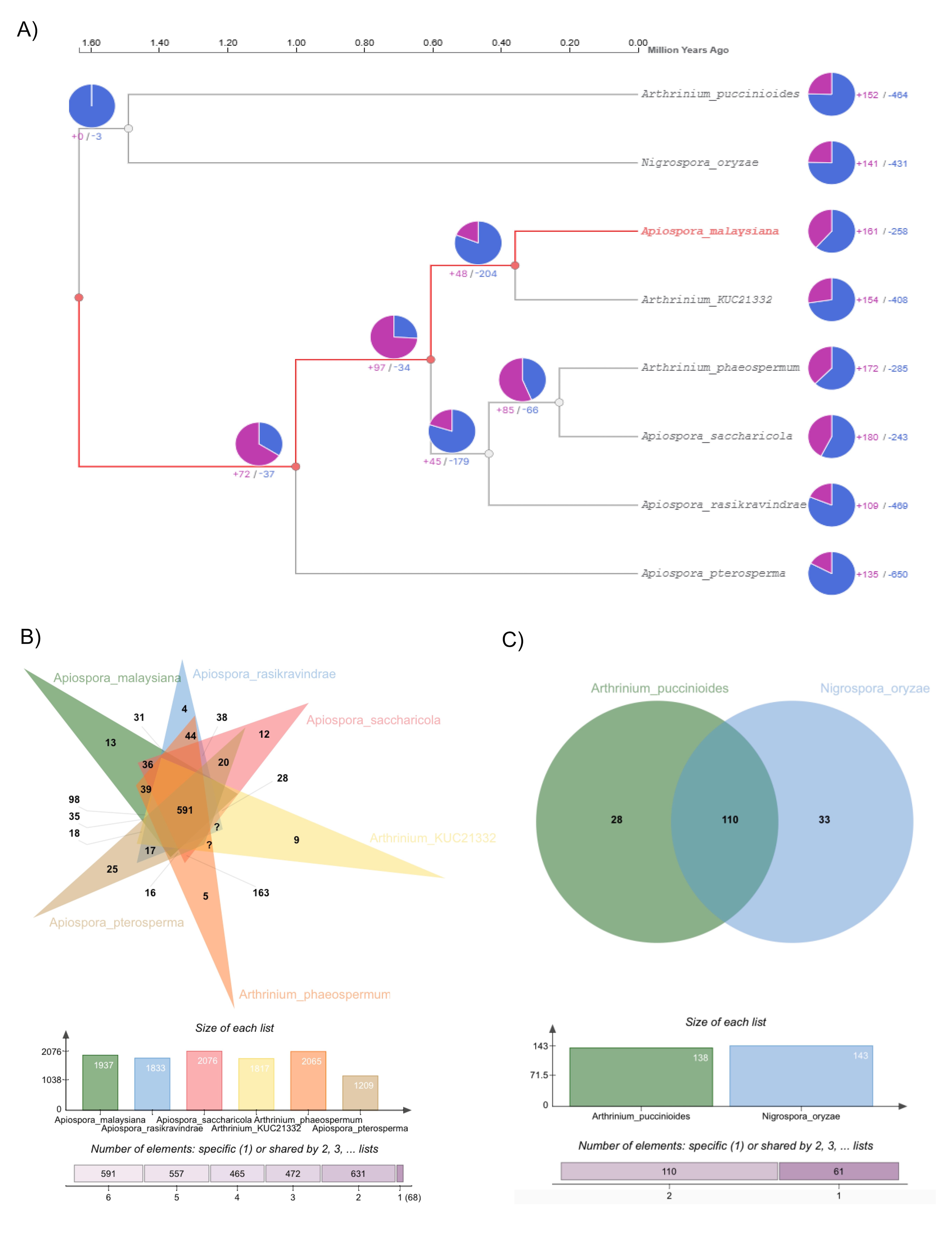

Supplement: dsaf011_suppl_Supplementary_Figure_S4 [file dsaf011_suppl_supplementary_figure_s4.jpeg]

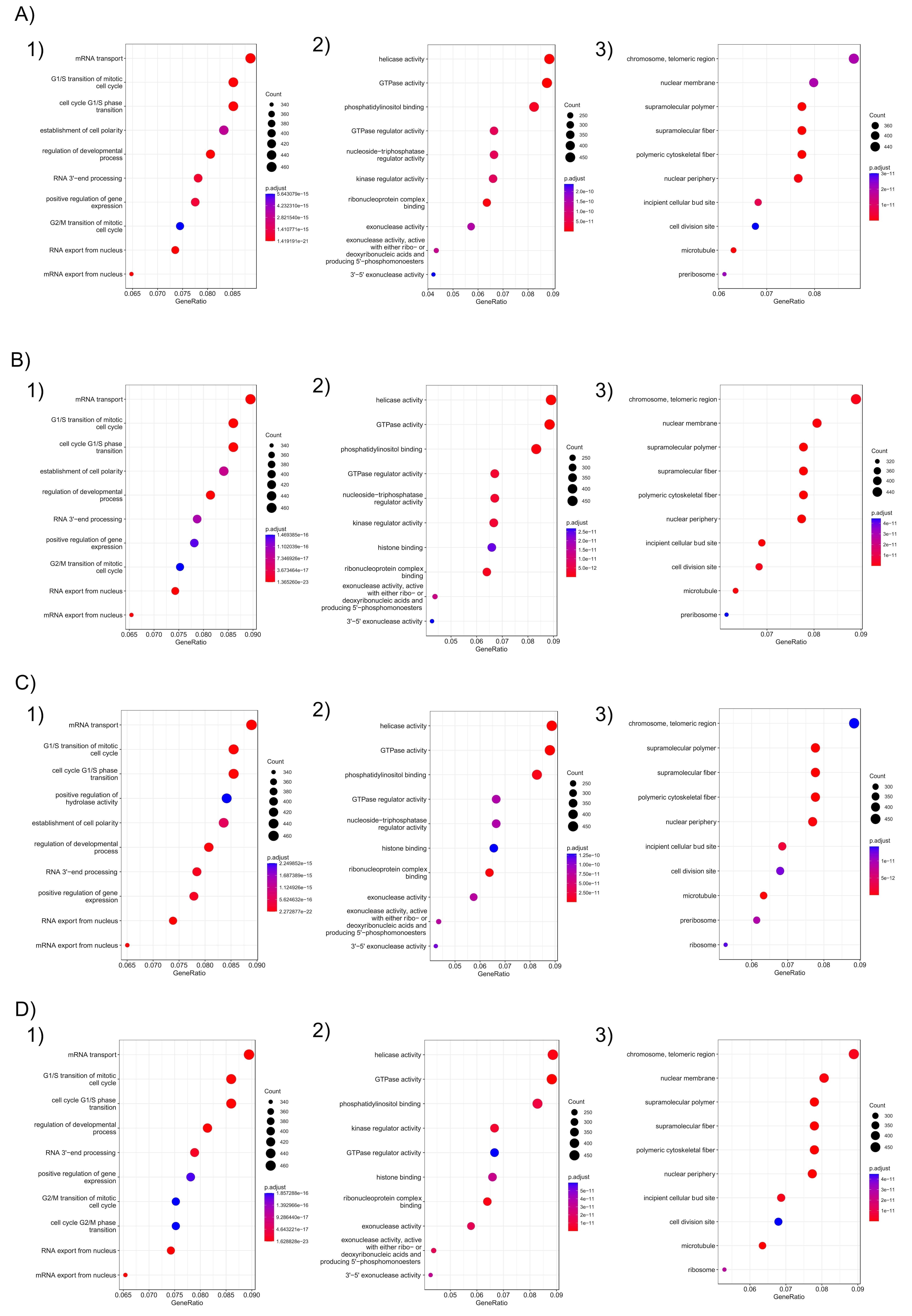

Supplement: dsaf011_suppl_Supplementary_Figure_S5_A [file dsaf011_suppl_supplementary_figure_s5_a.jpeg]

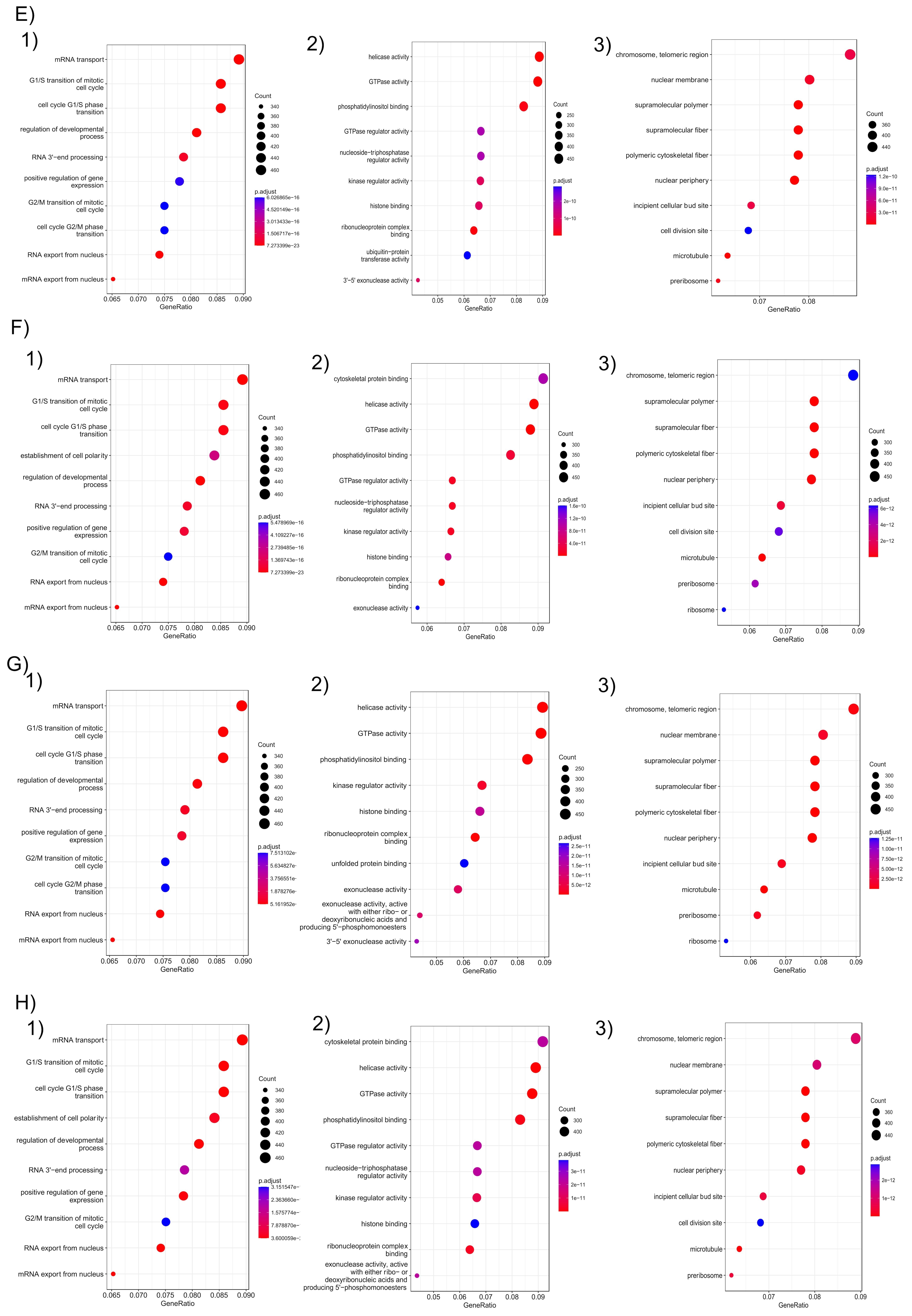

Supplement: dsaf011_suppl_Supplementary_Figure_S5_B [file dsaf011_suppl_supplementary_figure_s5_b.jpeg]

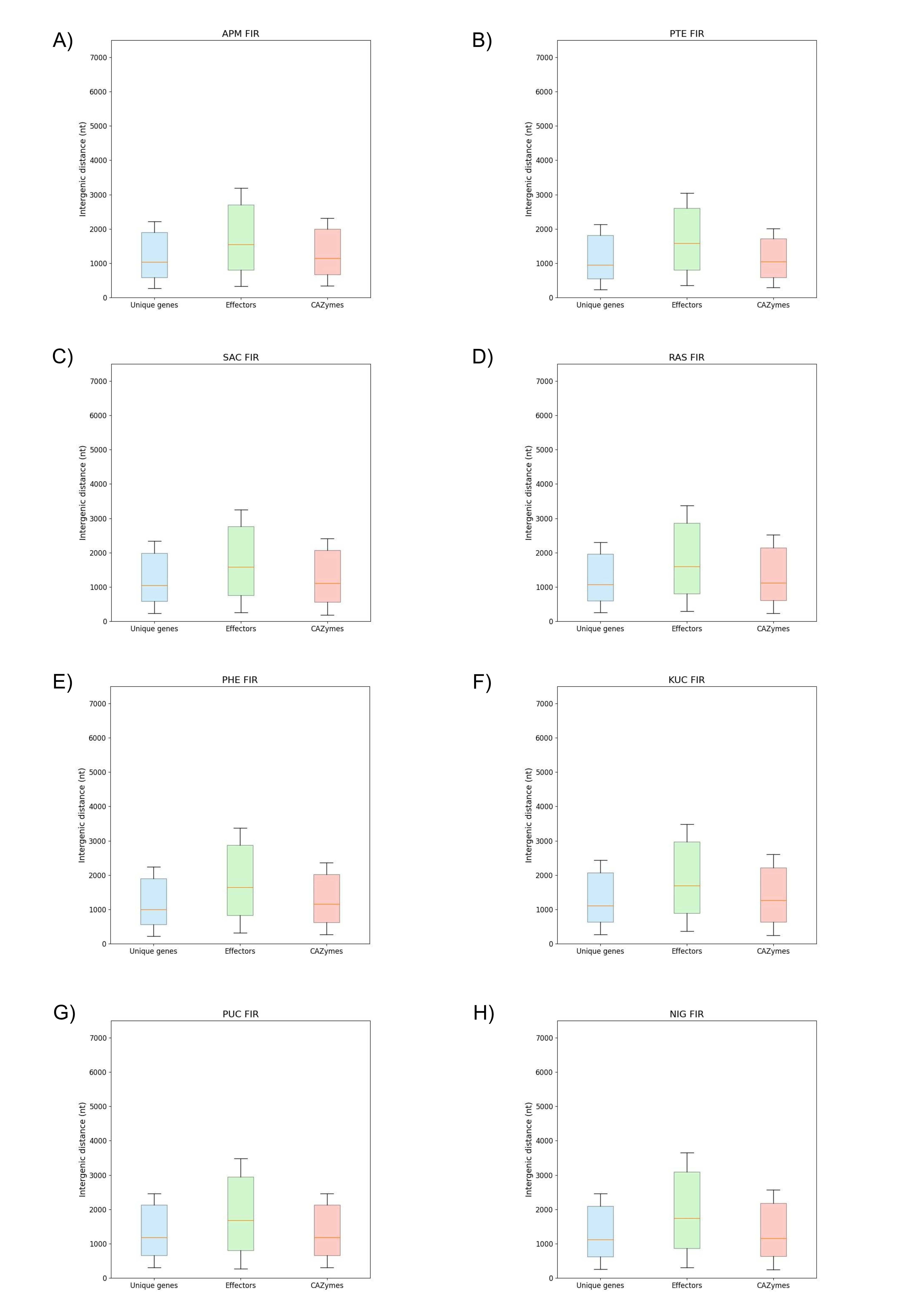

Supplement: dsaf011_suppl_Supplementary_Figure_S6 [file dsaf011_suppl_supplementary_figure_s6.jpeg]

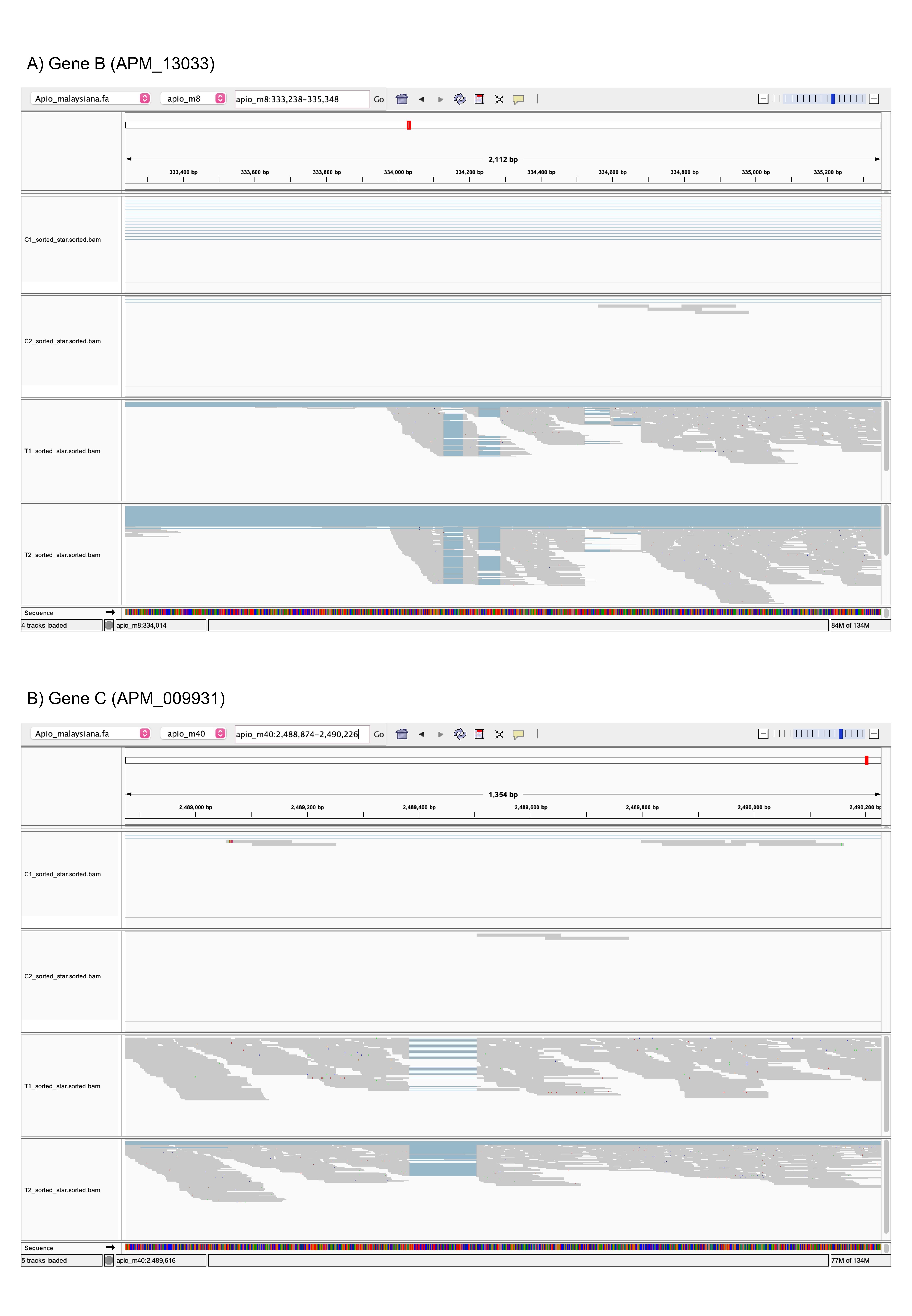

Supplement: dsaf011_suppl_Supplementary_Figure_S7 [file dsaf011_suppl_supplementary_figure_s7.jpeg]

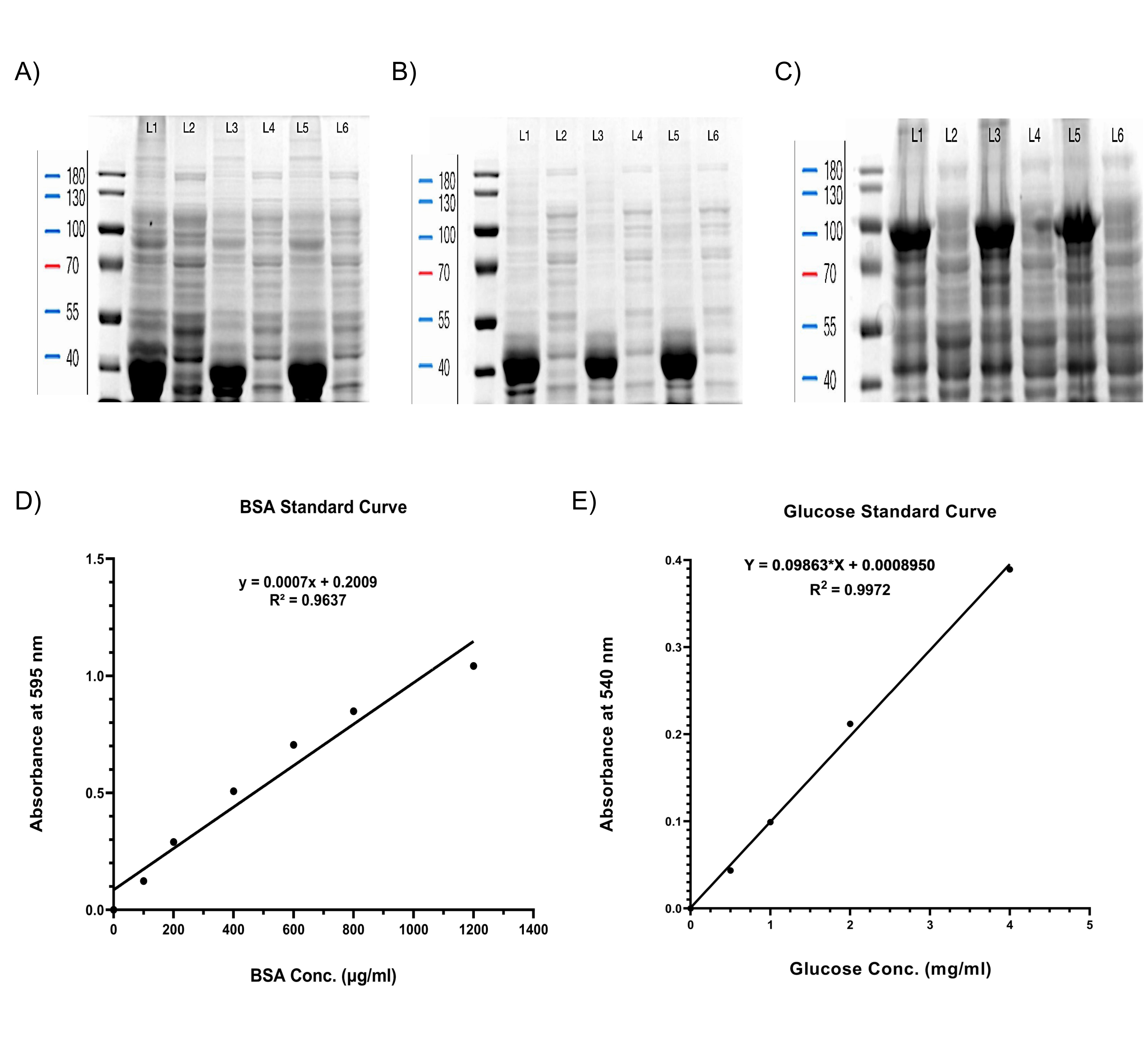

Supplement: dsaf011_suppl_Supplementary_Figure_S8 [file dsaf011_suppl_supplementary_figure_s8.jpeg]
