# Supplementary material for: Comparative genomics of endophytic fungi Apiospora malaysiana with related ascomycetes indicates adaptation attuned to lifestyle choices with potential sustainable cellulolytic activity
Source: DNA Res. 2025 May 10;32(3):dsaf011. doi: 10.1093/dnares/dsaf011 (PMC12202052; doi:10.1093/dnares/dsaf011)
Supplement: dsaf011_suppl_Supplementary_Figures_S1-S8 [file dsaf011_suppl_supplementary_figures_s1-s8.docx]

**SUPPLEMENTARY FIGURE LEGEND:**

**Fig S1**: Strain identification and annotation of the genome. A) PCR amplification of the ITS region. B) Heterozygosity of *A. malaysiana* with only one peak when plotted with coverage on X – axis and frequency on Y – axis, plotted using GenomeScope2*.* C) Top 10 metabolic pathways in *A. malaysiana* and their percentage of occupancies of genes among themselves. D) MAT locus of *A. malaysiana* (scaffold 40) with *A. KUC21332* (contig_10).

**Fig S2**: Heatmap representation of CAZyme family abundance across studied organisms. The heatmaps depict the distribution of (A) Glycoside Hydrolases (GH), (B) Glycosyl Transferases (GT), (C) Auxiliary Activities (AA), (D) Carbohydrate Esterases (CE), (E) Polysaccharide Lyases (PL), and (F) Carbohydrate-Binding Modules (CBM). Darker shades indicate higher gene counts within a given CAZyme family, with values displayed inside each cell.

**Fig S3**: Endophytic study, plants grown in MS media without (*control*) and with *A. malaysiana* (*treated*). A) Plant growth comparison in control and treated groups after two week of post inoculation in moong plants. B) Microscopic view of plant roots section of control and treated groups of moong plants at 100X. C) Plant growth comparison in control and treated groups after 12 days of post inoculation in rice plants. D) Microscopic view of plant roots section of control and treated groups of rice plants at 100X. (C and T represent control and treated plants samples respectively).

**Fig S4**: Emergence and relatedness of the organisms. Species emergence timelines in million years using orthologous genes clusters with expanded gene families in purple and contracted gene families in blue colour. A) Species emergence timelines in million years, analysed using CAFÉ with their genome expansion and contractions. B) 591 clusters of *Apiospora* and *Arthrinium* species, excluding known pathogens was shared. C) 110 Clusters were shared among pathogens (*A. puccinioides & N. oryzae*), generated by orthovenn3.

**Fig S5i &5ii**: Gene enrichment analysis of the eight species studied: A) *A. malaysiana.* B) *A. pterosperma.* C) *A. saccharicola.* D) *A. rasikravindrae.* E) *A. phaeospermum.* F) *A. KUC21332.* G) *A. puccinioides.* H) *N. oryzae* for: (1) Biological process (BP). (2) Molecular function (MF). (3) Cellular component (CC). Gene ratio is in X – axis and enriched process or function or components is represented in Y- axis for BP, MF and CC respectively. Dot size depicts count and colour for p adjustment shown right of each plot, generated by clusterProfiler of R.

**Fig S6**: Boxplot of intergenic distances for core genes, effectors, and CAZymes across all species. A) *A. malaysiana.* B) *A. pterosperma.* C) *A. saccharicola.* D) *A. rasikravindrae.* E) *A. phaeospermum.* F) *A. KUC21332.* G) *A. puccinioides.* H) *N. oryzae.* Blue box represents unique gene flanking intergenic distances, green represents effectors flanking intergenic distances, whereass pink represent CAZymes intergenic distances of each organism, with x -axis for genes and y – axis for their intergenic distances in number of nucleotides.

**Fig S7**: Expression analysis of cloned genes B (APM_13033) and gene C (APM_009931) visualized using Integrative Genomics Viewer (IGV). Mapped alignments are shown for control and treated samples across replicates, allowing for comparative assessment of gene expression patterns.

**Fig S8**: Expression of recombinant proteins. A) Gene 1. B) Gene 2. C) Gene 3 at 0.1 mM, 0.5 mM, and 1.0 mM IPTG. L1: 0.1 mM induced pellet, L2: 0.1 mM induced supernatant, L3: 0.5 mM induced pellet, L4: 0.5 mM induced supernatant, L5: 1.0 mM induced pellet, L6: 1.0 mM induced supernatant, run on 12 % SDS-PAGE. D) BSA standard curve (X-axis: Amount of BSA in µg; Y-axis: OD at 595 nm). E) Glucose standard curve (X - axis: Glucose concentration; Y - axis: Absorbance at 540 nm).
